# Analysis of oral health productivity in the Brazilian prison system from 2017 to 2022: a retrospective ecological study

**DOI:** 10.31744/einstein_journal/2025AO1476

**Published:** 2025-07-23

**Authors:** Artur Freitas Riccioppo, Naessa Santos Borges Zure, Lucas Gonçalves de Sousa, Pedro Henrique Mota Rodrigues, Thallys Rodrigues Félix, Álex Moreira Herval

**Affiliations:** 2 Universidade Federal de Uberlândia Uberlândia MG Brazil Universidade Federal de Uberlândia, Uberlândia, MG, Brazil.

**Keywords:** Oral health, Prisoners, Dentistry, Health services

## Abstract

**Objective:**

This study compared the productivity of prison oral health teams during the COVID-19 crisis (2020–2022) with the previous three-year period (2017–2019) to identify the number and type of dental procedures performed.

**Methods:**

We employed an ecological and retrospective study using Primary Care Health Information System data. We analyzed data from all municipalities that reported the productivity of Prison Primary Care Teams from 2017 to 2022, totaling 418 municipalities distributed across all five Brazilian regions. Data pertaining to 15 oral health procedures were collected and subsequently grouped into five categories: preventive, urgent, extraction, periodontics, and restorative. The data revealed a non-normal distribution and were evaluated using the Wilcoxon test.

**Results:**

The findings revealed inequality between regions, with the productivity of several municipalities being close to or equal to zero. Although the number of restorative procedures reduced in both periods (2017-2019 and 2020-2022), it was not statistically significant. However, statistically significant increases were observed, with the exception of restorative and periodontal procedures. Urgent and extraction procedures prevailed over restorative and periodontal treatments.

**Conclusion:**

Access to Brazilian oral health procedures for inmates is low and remained unaffected by the pandemic, maintaining similar characteristics with increased productivity during that period.

## In Brief

Riccioppo et al. analyzed dental productivity in prison units within Brazil from 2017 to 2022, disclosing an unequal provision of dental procedures among different Brazilian regions. Additionally, while preventive, urgent, and extraction procedures increased during the COVID-19 pandemic, periodontics and restorative procedures showed no significant change in pattern.

## Highlights

■ The quantity of dental procedures in prisons differs across Brazilian regions.
■ Prisoners’ access to dental procedures was limited even prior to the pandemic.
■ Preventive, urgent, and dental extraction procedures exhibited increases.
■ Restorative and periodontic procedures did not exhibit statistically significant changes.

## Graphical abstract

**Figure:**
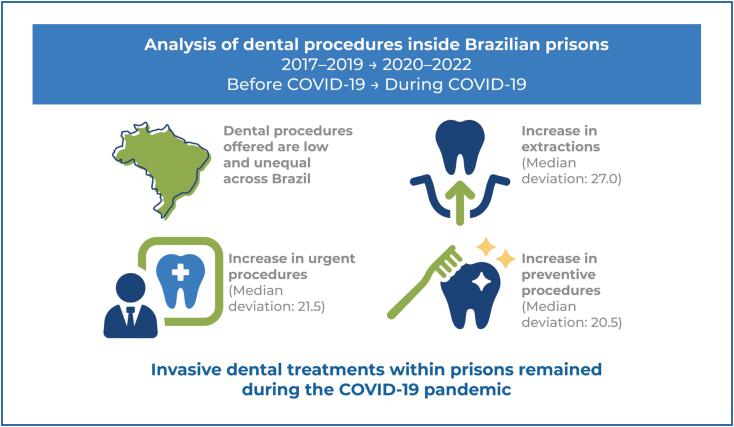

## INTRODUCTION

Inmates in Brazil are more susceptible to developing certain diseases owing to the infrastructure and organization of Brazilian prisons.^(1)^ Limited dental care access and poor hygiene conditions cause a higher prevalence of common oral problems, such as tooth decay, periodontal disease, tooth loss, and frequent painful episodes.^(2)^ Additionally, unhealthy diets, smoking, stress, and limited access to drinking water worsen the oral health of individuals deprived of liberty, whose health is usually already compromised by their lifestyles prior to detention.^(3,4)^

Despite specific policies to restructure healthcare in the Brazilian prison system, ensuring equitable access to oral health depends on overcoming challenges. Technical and political obstacles include deficient infrastructure of prison facilities, insufficient number of qualified human resources, and the lack of preventive programs, timely treatment, regular monitoring, and evaluation of services.^(5,6)^ Overcrowding, structural precariousness, insalubrity, violence, stigmatization, and discrimination lead to high degrees of stress, frustration, and demotivation among health professionals, limiting healthcare access for the prison population.^(7-9)^

Considering these sanitary and structural weaknesses, Brazilian prisons were considered high-risk scenarios for contagion of COVID-19 virus, necessitating the adoption of protective strategies designed for people deprived of liberty, prison officers, and other professionals.^(1,10)^ Although prison units represent a reality of social isolation, overcrowding has made it hard to implement social distancing measures.^(11)^

## OBJECTIVE

This study aimed to analyze the productivity of dental procedures in Brazilian prisons between 2017 and 2022. It hypothesized that oral health procedures in prison health units reduced during the three years influenced by COVID-19 containment measures (2020–2022) compared to the three years preceding the pandemic (2017–2019).

## METHODS

### Study design and ethical aspects

This study employed a retrospective observational design using public data from 2017 to 2022. The analysis units were Brazilian municipalities with Prison Primary Care Teams that reported dental productivity to the Ministry of Health. The study sought to answer the following question: Did the productivity of oral health teams in the Brazilian prison system reduce during the COVID-19 crisis? Because this study used publicly accessible data, it did not submit to a research ethics committee, agreeing with Resolution 510 of April 7, 2016, of the National Health Council of the Brazilian Ministry of Health.

### Background and study scope

The study included all Brazilian municipalities that presented dental procedures performed by oral health teams within the prison system between 2017 and 2022. The Brazilian prison system faces significant challenges in different regions. During the study, data from the state secretariats responsible for prison units reported a prison population of 644,316 individuals, despite the total capacity of facilities being 488,035, resulting in a deficit of 156,281 places. The Brazilian prison system includes 840 dental practices, 968 dentists, and 545 oral health technicians/assistants distributed among 1,388 prison facilities to meet the oral health needs of this population.^(12)^

### Access and data collection

Dental production records were collected from the Primary Care Health Information System (SISAB - *Sistema de Informação em Saúde para a Atenção Básica*) database supported by the Department of Information Technology of the Unified Health System (DATASUS - *Departamento de Informação e Informática do SUS*) of the Brazilian Ministry of Health.^(13)^ An established collection protocol included filling in the available fields on the website, following the standard sequence: "geographical unit" - Brazil; "jurisdiction" - January 2017 to December 2022 (representing the periods before, during, and after the COVID-19 pandemic); "report line" - municipality; "report column" - OH procedure (representing oral health procedures); "team type" - Prison Primary Care Team (EABP - *Equipe de Atenção Básica Prisional*); "professional category" - dental surgeon. The "age group," "sex," "place of assistance," and "care type" items were ignored. Dental care was selected as the "production type" item, and "consultation type" was not assigned.

Under "procedures," the following 15 dental procedures were deemed relevant for data collection: pulp access and medication (per tooth), pulp capping, dental dressing with or without preparation, abscess drainage, bacterial plaque disclosing, permanent tooth extraction, oral hygiene counseling, dental pulpotomy, subgingival scraping (per sextant), supragingival scraping (per sextant), bacterial plaque removal, anterior permanent tooth restoration, posterior permanent tooth restoration, temporary cavity sealing, and alveolitis treatment.

### Treatment of variables

All collected procedures were considered numerical variables and linked to each performed dental treatment by year and municipality. The procedures were grouped into five categories according to type: preventive (bacterial plaque disclosing, oral hygiene counseling, and bacterial plaque removal), urgent (pulp access and medication, abscess drainage, alveolitis treatment, dental pulpotomy, and dental dressing with or without preparation), extraction (permanent tooth extraction), periodontics (subgingival and supragingival scraping), and restorative (anterior and posterior permanent tooth restorations, temporary cavity sealing, and pulp capping).

### Data analysis

All data were exported from the national database to a Microsoft Excel^®^ spreadsheet, where they were tabulated and imported into Jamovi^®^ software. A descriptive analysis first considered central tendency measures and then verified the assumptions of normality. Non-normal distribution was observed, and the Wilcoxon test was employed to compare the three-year periods.

## RESULTS

This study analyzed the data on dental productivity from Prison Primary Care Teams in 418 Brazilian municipalities. The distribution of municipalities by Brazilian regions was 31 (7.4%) in the north, 108 (25.8%) in the northeast, 103 (24.6%) in the southeast, 69 (16.5%) in the south, and 107 (25.6%) in the central-west.

A descriptive analysis revealed the procedures performed by Prison Primary Care Teams from 2017 to 2019, with a mean of 283.7 (±109.9) preventive procedures, 78.4 (±360) urgent procedures, 111.1 (±326) extractions, 68.7 (±208) periodontal treatments, and 158.2 (±366) restorations. From 2020 to 2022, these means were 364.1 (±1137), 116.5 (±318), 161.7 (±338), 66.4 (±205), and 154.4 (±364), respectively.

Table 1 reveals the mean, standard deviation, median, and range of the procedures analyzed in each Brazilian region. The north region had the highest mean of preventive procedures and extractions in both analyzed periods. For periodontics, the north region had the highest mean in the first period (2017-2019) and the lowest in the second (2020-2022) compared to other areas. Urgent and restorative procedures exhibited the highest means in both periods for teams in the south. Regarding the median and range, all procedures and regions had a low median compared to a broad data range, indicating a high concentration of municipalities with productivity records close to or equal to zero.

**Table 1:** Descriptive analysis of dental productivity of the three-year periods (2017-2019 and 2020-2022) among Brazilian regions

Procedure	Three-year period	Brazilian Region					
		Measurement	Central-west	Southeast	Northeast	South	North
Preventive	2017–2019	Mean ± SD	118 ± 527	162±447	287±620	412±1917	720±1575
		Median (range)	20 (4141)	1 (3325)	51.5 (3725)	5 (15402)	113 (7860)
	2020–2022	Mean ± SD	211±339	524±1620	328±744	336±1605	548±796
		Median (range)	44 (1574)	49 (13844)	42.5 (5325)	16 (13156)	75 (2805)
Urgent	2017–2019	Mean ± SD	56.9±183	76.2±219	45.8±196	142±708	132±485
		Median (range)	3 (1433)	2 (1619)	2 (1972)	2 (5834)	6 (2670)
	2020–2022	Mean ± SD	113±277	160±438	58.8±118	168±414	68.0±117
		Median (range)	31 (1920)	31 (3942)	9 (774)	33 (3064)	20 (469)
Extraction	2017–2019	Mean ± SD	80.7±294	69.6±159	101±191	163±420	274±719
		Median (range)	8 (2932)	5 (918)	12 (873)	14 (2961)	30 (3697)
	2020–2022	Mean ± SD	127±387	176±340	140±239	183±333	264±443
		Median (range)	35 (3526)	59 (1842)	41.5 (1260)	53 (2081)	81 (1919)
Periodontal	2017–2019	Mean ± SD	58.9±160	36.4±95.1	52.1±121	129±369	135±315
		Median (range)	6 (1371)	0 (521)	5.50 (990)	4 (2201)	2 (1251)
	2020–2022	Mean ± SD	50.4±163	93.4±293	51.4±94.3	83.2±264	46.8±72.1
		Median (range)	7 (1561)	11 (2186)	5.50 (407)	6 (1938)	3 (259)
Restorative	2017–2019	Mean ± SD	166±346	112±235	102±171	273±610	227±497
		Median (range)	22 (2486)	2 (1610)	24.0 (951)	47 (3500)	14 (1862)
	2020–2022	Mean ± SD	143±314	195±487	64.3±109	276±492	103±153
		Median (range)	43 (2430)	51 (3258)	15.5 (522)	83 (2419)	29 (604)

Source: Brazil. Ministério da Saúde. Sistema de Informação em Saúde para Atenção Básica. Brasília (DF): Ministério da Saúde; 2023 [citado 2024 Jun 17]. Disponível em: https://sisab.saude.gov.br/paginas/acessoRestrito/relatorio/federal/saude/RelSauProducao.xhtml^(13)^

SD: standard deviation.

Table 2 depicts the comparative analysis of the mean number of procedures between 2017–2019 and 2020–2022. All treatments, except restorations and periodontics, revealed a statistically significant increase. Only restorations demonstrated reductions but without statistical significance.

**Table 2:** Comparative analysis of the productivity by dental procedure groupings between the analyzed periods (2017–2019 and 2020–2022)

Procedure	Mean deviation	Productivity of prison health teams		
		Tendency	Confidence interval (Lower-Upper)	p value
Preventive	20.5	Increase	7.0 – 41.0	0.002
Urgent	21.5	Increase	14.5 – 30.5	<0.001
Extraction	27.0	Increase	18.5 – 37.0	<0.001
Periodontics	3.6	Increase	-4.5 – 4.0	0.921
Restorative	-3.6	Reduction	-14.0 – 10.0	0.714
Total	81.5	Increase	35.5 – 132.0	<0.001

Source: Brazil. Ministério da Saúde. Sistema de Informação em Saúde para Atenção Básica. Brasília (DF): Ministério da Saúde; 2023 [citado 2024 Jun 17]. Disponível em: https://sisab.saude.gov.br/paginas/acessoRestrito/relatorio/federal/saude/RelSauProducao.xhtml^(13)^

## DISCUSSION

The analysis of the two studied periods (2017–2019 and 2020–2022) revealed that the COVID-19 pandemic did not reduce the productivity of the prison health teams. However, numerous municipalities without productivity records may have influenced this result. Although elective treatments were suspended at times during the health crisis, the three-year analysis revealed a statistically significant increase in urgent procedures and extractions over restorative and periodontal treatments, indicating an aggravation of invasive procedures for this population.

Other studies have reported the pattern of invasive treatments performed in prison units, demonstrating that the health surveillance model recommended by the Brazilian Unified Health System has not advanced in prison dentistry.^(14)^ Research among the prison population in the metropolitan region of Salvador (Bahia - northeast region of Brazil) revealed high rates of cavities and tooth loss compared to a low percentage of restored teeth.^(15)^ Similarly, in Pará (north region), extraction is the most frequently performed dental procedure (63.34%), highlighting the need to expand efforts to prevent tooth loss and incorporate the rehabilitation of existing damage.^(2)^ Although prison units lack preventive dental care,^(15)^ preventive treatments exhibited an increase when comparing the studied periods.

It is noteworthy that the oral condition of individuals deprived of liberty reveals a high incidence of common oral diseases. Oliveira et al^(14)^ observed a high prevalence of decay, often causing painful symptoms and abscesses. Motghare et al.^(16)^ discovered that the majority of the prison population (92.3%) had periodontal disease, which worsens as the sentence length extends. Despite the harmful effects on oral health, frequent tobacco and narcotic consumption before imprisonment may mask the pain for this population. However, dental pain episodes increase when these individuals enter the prison system and cannot use illicit drugs.^(4)^

A study in Kuwait revealed that the pandemic affected the dental specialty supply differently, with significant reductions in orthodontics, endodontics, and periodontics and a mild increase in pediatric dentistry and surgical procedures.^(17)^ In Poland, preventive and restorative dental procedures decreased sharply at the onset of the pandemic, while urgent and surgical procedures increased.^(18)^ The limited access to prison health services in the United Kingdom worsened pre-existing conditions and led to the emergence of new diseases, increasing health requirements.^(19)^ International data corroborate the reality of Brazilian prisons, with an increase in urgent procedures and extractions in the three years affected by the pandemic.

Urgent dental care provided by the Brazilian public health system decreased considerably because of the pandemic, despite not been suspended.^(4)^ Dental surgeries performed in the public system also dropped substantially in the first year of the pandemic, recording a 68.6% reduction in the southeast region.^(20)^ In addition, the recommendation to reduce elective procedures and consultations at certain times may have changed the previous care profile.^(21)^ The present study differs from others in the Brazilian scenario, as it indicates an increase in the number of procedures, potentially related to the extended period analyzed and the possible measures to manage the suppressed demand in 2021 and 2022 owing to the COVID-19 pandemic.

The descriptive findings revealed several municipalities with low productivity in the first three years analyzed (medians close to or equal to zero), corroborating Brazilian studies stating that prison health teams had low productivity even before the pandemic. Lôbo et al.^(3)^ highlighted the high monthly variability in consultations and the irregular dental care productivity in the Brazilian prison system even after the increase of health teams since the implementation of the national policy, indicating a scarce and repressed dental service supply. The unpredictable variation in these care patterns within the prison system compromises health action planning, generating uncertainty regarding the sector's productive capacity.^(3)^

Queiroz et al.^(22)^ reviewed studies on healthcare in the prison system, highlighting that the primary approach to inmate assistance is episodic and treatment-oriented care, with health promotion and preventive actions implemented intermittently. The existing difficulties were also aggravated by insufficient communication between the health and justice systems and the limitations pertaining to the training of health professionals working with inmates.

Oliveira et al^(14)^ addressed oral health in a prison unit in Rio de Janeiro, highlighting the predominance of curative and invasive treatments—mainly dental extractions—which reveal a limited approach related to public health principles and the integrality of the Brazilian Unified Health System. Furthermore, they stressed the urgency of increasing the number of dental professionals to guarantee humanized and quality care.

Overcrowding and the precarious structural condition of prison facilities exacerbate the challenges of prison healthcare, fostering the extensive spread of diseases. Assistance gaps, chronic shortages of human resources, the lack of urgent care, and insufficient funds for dental services also persist. These challenges endorse the pressing need for structural reforms and funding to ensure efficient healthcare for the prison population.^(23)^

A possible limitation of this study is the use of secondary sources whose data are recorded by health teams directly into the national information system DATASUS. These sources may present information biases owing to possible errors upon recording (under or overreporting data). Despite this limitation, the data available in DATASUS are crucial for health action planning and decision making to strengthen the ability to manage, evaluate, and monitor actions, such as in the present study.

## CONCLUSION

The provision of oral health procedures for inmates is low and unequal among Brazilian regions. The pandemic did not reduce the productivity of prison oral health teams, as initially hypothesized. A statistically significant increase in productivity was observed during the COVID-19 crisis, and the invasive pattern of prison dentistry procedures remained.
